# Identification of crosstalk genes and immune characteristics between Alzheimer’s disease and atherosclerosis

**DOI:** 10.3389/fimmu.2024.1443464

**Published:** 2024-08-12

**Authors:** Wenhao An, Jiajun Zhou, Zhiqiang Qiu, Peishen Wang, Xinye Han, Yanwen Cheng, Zi He, Yihua An, Shouwei Li

**Affiliations:** ^1^ Department of Neurosurgery, Sanbo Brain Hospital, Capital Medical University, Beijing, China; ^2^ Department of Research and Development, Beijing Yihua Biotechnology Co., Ltd, Beijing, China

**Keywords:** Alzheimer’s disease, atherosclerosis, crosstalk genes, bioinformatics analysis, immunology

## Abstract

**Background:**

Advancements in modern medicine have extended human lifespan, but they have also led to an increase in age-related diseases such as Alzheimer’s disease (AD) and atherosclerosis (AS). Growing research evidence indicates a close connection between these two conditions.

**Methods:**

We downloaded four gene expression datasets related to AD and AS from the Gene Expression Omnibus (GEO) database (GSE33000, GSE100927, GSE44770, and GSE43292) and performed differential gene expression (DEGs) analysis using the R package “limma”. Through Weighted gene correlation network analysis (WGCNA), we selected the gene modules most relevant to the diseases and intersected them with the DEGs to identify crosstalk genes (CGs) between AD and AS. Subsequently, we conducted functional enrichment analysis of the CGs using DAVID. To screen for potential diagnostic genes, we applied the least absolute shrinkage and selection operator (LASSO) regression and constructed a logistic regression model for disease prediction. We established a protein-protein interaction (PPI) network using STRING (https://cn.string-db.org/) and Cytoscape and analyzed immune cell infiltration using the CIBERSORT algorithm. Additionally, NetworkAnalyst (http://www.networkanalyst.ca) was utilized for gene regulation and interaction analysis, and consensus clustering was employed to determine disease subtypes. All statistical analyses and visualizations were performed using various R packages, with a significance level set at p<0.05.

**Results:**

Through intersection analysis of disease-associated gene modules identified by DEGs and WGCNA, we identified a total of 31 CGs co-existing between AD and AS, with their biological functions primarily associated with immune pathways. LASSO analysis helped us identify three genes (C1QA, MT1M, and RAMP1) as optimal diagnostic CGs for AD and AS. Based on this, we constructed predictive models for both diseases, whose accuracy was validated by external databases. By establishing a PPI network and employing four topological algorithms, we identified four hub genes (C1QB, CSF1R, TYROBP, and FCER1G) within the CGs, closely related to immune cell infiltration. NetworkAnalyst further revealed the regulatory networks of these hub genes. Finally, defining C1 and C2 subtypes for AD and AS respectively based on the expression profiles of CGs, we found the C2 subtype exhibited immune overactivation.

**Conclusion:**

This study utilized gene expression matrices and various algorithms to explore the potential links between AD and AS. The identification of CGs revealed interactions between these two diseases, with immune and inflammatory imbalances playing crucial roles in their onset and progression. We hope these findings will provide valuable insights for future research on AD and AS.

## 1 Introduction

With the continuous progress of modern society and medical technology, human life expectancy is steadily increasing, which is a delightful development (1–3). However, this brings along a series of challenges, one of which is the rise in age-related diseases (4). Among these ailments, Alzheimer’s disease and atherosclerosis stand out as two significant focal points. Alzheimer’s disease leads to cognitive decline, while atherosclerosis triggers cardiovascular diseases, causing immense suffering not only to the patients themselves but also imposing a heavy burden on their families and society at large (5, 6).

Alzheimer’s disease (AD) is a progressive neurodegenerative disorder, typically characterized by memory loss, cognitive decline, and behavioral abnormalities (7). Currently, it affects a significant number of individuals globally, with a growing trend. Data shows that in the United States alone, there are approximately 6.7 million AD patients aged 65 and older, and this number is projected to exceed 13.8 million by 2060 (8). The exact cause of AD remains unclear, but research suggests that genetic factors, abnormal protein metabolism, and neuroinflammation may be involved in its pathogenesis, with neuronal death and abnormal protein accumulation likely playing significant roles (9, 10). Initial symptoms of AD typically include mild memory issues, such as forgetting important dates or events, progressing to severe memory loss and the inability to navigate familiar surroundings (11). As the disease progresses, patients may also experience language impairments, mood swings, social withdrawal, and other symptoms (12). Currently, there is no cure for Alzheimer’s disease, but some medications and non-pharmacological therapies can help slow disease progression and alleviate symptoms (13). Therefore, early diagnosis and intervention are crucial for managing this condition.

Atherosclerosis (AS) is a chronic, progressive arterial disease characterized by the deposition of lipid plaques within the blood vessel walls and thickening of the vessel walls (14). Rough estimates suggest that currently there are billions of individuals globally afflicted by this condition (15). The primary causes of AS include dyslipidemia, chronic inflammatory responses, and endothelial dysfunction within the blood vessels, among other factors (16). This disease has a wide-ranging impact, affecting various arteries throughout the body, including those of the heart, carotid, cerebral, and peripheral arteries, leading to cardiovascular diseases such as myocardial infarction, and cerebrovascular diseases such as stroke, making it a leading cause of death worldwide (17–19). When confronting AS, prevention, early diagnosis, and aggressive treatment are often paramount in controlling and managing the disease (20).

Advancements in molecular biology and genomics have significantly propelled scientists’ understanding of the genetic basis of complex diseases (21, 22). Gene expression represents the activity level of specific genes at particular times and under specific conditions, determining cellular characteristics and functions, and largely influencing the health and disease states of an organism (23). By studying gene expression, we can discern which genes are activated or suppressed under certain conditions, thus unveiling the biochemical reactions and signal transduction processes within cells (24). Transcriptomic analysis, by comprehensively examining all mRNA expression in a cell or tissue, provides a systematic understanding of gene expression regulatory networks (25). This research approach not only allows for the quantitative analysis of the expression levels of tens of thousands of genes but also captures the interactions and regulatory relationships between different genes. It holds significant importance in revealing gene expression differences under various physiological states and disease conditions, aiding scientists in identifying potential pathogenic genes and biomarkers (26, 27).

Currently, there is increasing evidence suggesting a close association between Alzheimer’s disease (AD) and atherosclerosis (AS) (28–30). They both belong to age-related diseases and are largely regulated by the immune system. Recent advancements in AD research have confirmed the significant role of peripheral immune dysfunction in its pathogenesis (31–33). Meanwhile, vascular aging and endothelial dysfunction also appear to be common triggers between the two diseases (34). However, there is relatively limited research on the correlation between these two diseases based on gene expression levels. To address this gap, we conducted in-depth analysis utilizing public online databases, involving 888 patients with either AD or AS, along with their corresponding healthy population. Our study aimed to explore the association between these two diseases, striving to identify shared crosstalk genes (CGs) and analyze the primary biological effects of these genes. Through the application of machine learning algorithms, we successfully identified the optimal diagnostic genes and hub genes shared by AD and AS. This finding was supported by consistent expression patterns across four databases, validating the accuracy of our results. Furthermore, we delved into understanding these two diseases through immune infiltration analysis and confirmed the associated networks of key genes. Finally, we successfully identified subtypes of these two diseases using CGs. In conclusion, through this study, we aim to provide new insights for future researchers in predicting these two diseases and exploring the mechanisms underlying their association.

## 2 Materials and methods

### 2.1 Data download and processing

We retrieved four gene expression datasets (GSE33000, GSE100927, GSE44770, and GSE43292) from the GEO database (https://www.ncbi.nlm.nih.gov/geo/). Among these, GSE33000 and GSE100927 were analyzed as disease experimental groups. The former includes 310 samples of Alzheimer’s disease patients and 157 samples of normal brain tissue, while the latter comprises 69 samples of atherosclerosis patients and 35 samples of normal arterial tissue. On the other hand, GSE44770 and GSE43292 were analyzed as disease validation groups. The former consists of 80 samples of Alzheimer’s symptomatic patients and 173 samples of normal brain tissue, whereas the latter includes 32 samples of atherosclerosis patients and 32 samples of normal arterial tissue. All gene expression datasets underwent standardization using the “normalizeBetweenArrays” package in R software.

### 2.2 Differential gene expression analysis

We utilized the “limma” package in R software to perform differential gene expression analysis on the standardized GSE33000 and GSE100927 datasets. For the GSE33000 dataset, the criteria for screening differential expression genes (DEGs) were set as |logFC|≥0.5 and p-value<0.05, while for the GSE100927 dataset, the criteria were |logFC|≥1 and p-value<0.05. Using volcano plots, we displayed the DEGs that met these criteria, highlighting genes with |logFC|≥1 in AD and |logFC|≥2 in AS. Additionally, gene expression heatmaps were generated to illustrate the top 30 upregulated or downregulated DEGs.

### 2.3 Weighted gene correlation network analysis identifies disease-related gene modules

Weighted gene correlation network analysis (WGCNA) is employed to discover highly correlated gene clusters (modules), and these modules are associated with external sample features and other modules through a module feature gene network approach (35). We utilized the “WGCNA” package in R software to construct the gene co-expression network. Firstly, the quality of samples and genes was inspected to ensure data quality met the requirements. Secondly, hierarchical clustering was performed on samples to detect outlier samples, and outliers were removed based on corresponding high values. Thirdly, the pickSoftThreshold function was used to compute an appropriate soft threshold, and a biologically meaningful scale-free network was established. Fourthly, the dynamic tree-cutting algorithm was employed to construct a topological overlap matrix, establish the gene co-expression network, and identify gene modules. Fifthly, by computing gene significance and module membership, gene modules were linked to clinical features, and the structure and associations of feature gene networks were visualized. Finally, genes from the respective modules were selected for subsequent analysis.

### 2.4 Identification and enrichment analysis of crosstalk genes

DEGs identified from GSE33000 and GSE100927, as well as gene modules obtained from WGCNA analysis from both datasets, were analyzed by taking their intersection. Overlapping genes were considered as crosstalk genes (CGs) related to both diseases. The CGs were uploaded to https://david.ncifcrf.gov/ for Gene Ontology (GO) analysis and Kyoto Encyclopedia of Genes and Genomes (KEGG) analysis. Bubble plot tools were utilized to visually represent the results. Relevant immune processes were selected from the c5.all.v7.5.1 gene set, and Gene Set Variation Analysis (GSVA) from the R package “GSVA” was used to calculate enrichment scores for each patient. Heatmaps were generated using the “pheatmap” package for visualization.

### 2.5 Filtering potential diagnostic genes in CGs

The Least Absolute Shrinkage and Selection Operator (LASSO) is a regularization method for linear regression. It adds an L1 regularization term to the loss function of the regression model to limit the sum of the absolute values of the model parameters. This allows many model parameters to become zero, achieving the goal of feature selection and model sparsity (36). We used 5-fold cross-validation to determine the optimal regularization parameter for AD (GSE33000) and AS (GSE100927) respectively, and analyzed the two databases using the “glmnet” package. Ultimately, we selected their intersection as the best diagnostic genes in the CGs of the two diseases. The expression patterns of these genes in the two diseases were displayed through box plots, and their diagnostic effectiveness was observed by the area under the receiver operating characteristic (ROC) curve (37). We also included datasets GSE44770 and GSE43292 for validation.

### 2.6 Building disease prediction model based on diagnostic genes

We utilized the “Irm” package in R software to incorporate the three identified optimal diagnostic genes and constructed a logistic regression model for predicting the occurrence of the related disease, generating a nomogram (38). “Scores” represent the scoring situation of each identified gene, while “Total Score” indicates the sum of scores for each gene. The accuracy of the model in disease prediction was evaluated through ROC curves, while calibration curves and decision curves were employed to assess the consistency between prediction and actual observation, incorporating the corresponding validation group for comprehensive model evaluation.

### 2.7 Construction of PPI network and screening of hub CGs

The construction and analysis of PPI networks help uncover the interactions between important proteins underlying diseases, thereby inferring key functions and pathways in disease progression (39). We utilized the online analysis tool STRING (https://string-db.org/) to compute PPI networks of CGs and visualized the results using Cytoscape software. In the process of screening hub CGs, the cytoHubba plugin was employed, along with four topological analysis methods, including Maximal Clique Centrality (MCC), Degree, Maximum Neighborhood Component (MNC), and Edge Percolated Component (EPC), to jointly identify hub expression genes. The expression profiles of hub expression genes across four databases were demonstrated using violin plots.

### 2.8 Analysis of immune cell infiltration

Immune cells exhibit specific patterns of infiltration and residence during the onset and progression of diseases. These patterns provide crucial clues and guidance for understanding their roles in disease mechanisms and offer key information for the development of novel therapeutic approaches (40). Utilizing tissue-based gene expression matrices, we employed the CIBERSORT algorithm to compute the infiltration levels of 24 immune cell types. Through box plots, stacked bar charts, and correlation heatmaps, we presented the infiltration results of immune cells along with their associated features.

### 2.9 Gene regulation and network analysis of interactions with diseases, drugs, and chemical substances

NetworkAnalyst (http://www.networkanalyst.ca) is an online platform used for complex meta-analysis of gene expression (41). In this study, we utilized the NetworkAnalyst platform for multifaceted analyses. Construction of the Gene-miRNA interaction network was based on the TarBase v8.0 database, while the TF-Gene interaction network relied on the ChEA database, and the study of TF-miRNA crosstalk was based on the RegNetwork database. Additionally, we employed the DisGeNET database, DrugBank database, and Comparative Toxicogenomics Database (CTD) to analyze associations between genes and diseases, interactions between proteins and drugs, and interactions between proteins and chemical substances.

### 2.10 Consensus clustering analysis identifies disease subtypes associated with CGs

The consensus clustering method is an unsupervised algorithm that effectively distinguishes different subtypes or subgroups within a dataset by identifying and clustering individual samples. Using CGs, we employed the Pam algorithm from the “ConsensusClusterPlus” package to determine subtypes for both AD and AS (42). Subsequently, through immune infiltration analysis and the GSVA algorithm, we analyzed the relevant features of the subtypes for these two diseases separately and presented these results using box plots and heatmaps.

### 2.11 Statistical analysis and visualization processing

This study employed R software (version 4.2.3, Windows platform), in conjunction with various software packages, for statistical analysis and plotting. To assess significant differences between two groups of data, we utilized two-sided Wilcoxon tests for analysis; while for evaluating correlations between two groups of data, Pearson correlation analysis was employed. In statistical terms, we defined a p-value less than 0.05 as having significance.

## 3 Result

### 3.1 Identification of DEGs in AD and AS

**Figure 1:**
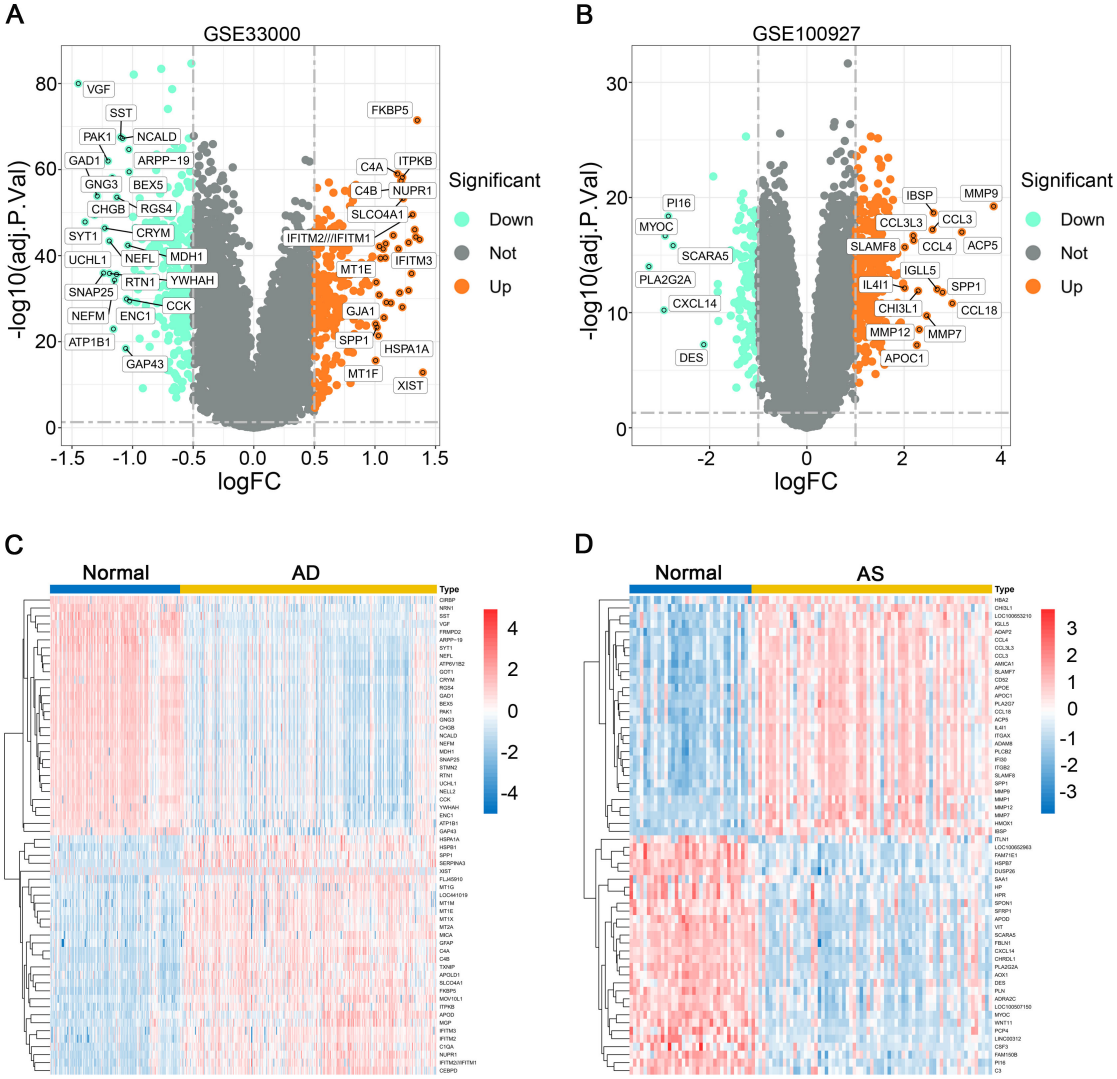
Differential gene expression analysis. **(A, B)** Volcano plots depict the differential expression genes (DEGs) in GSE33000 and GSE100927. **(C, D)** Heatmaps illustrate the expression patterns of corresponding DEGs in GSE33000 and GSE100927.

After standardizing the required datasets, we identified 550 DEGs (**Supplementary Table 1**) in the AD dataset GSE33000, comprising 252 upregulated genes and 298 downregulated genes. In the AS dataset GSE100927, we identified 463 DEGs (**Supplementary Table 2**), including 326 upregulated genes and 137 downregulated genes. The volcano plots illustrate all DEGs in AD and AS (**Figures 1A, B**), while the heatmap displays the top 30 upregulated or downregulated DEGs with the highest differences between the two diseases (**Figures 1C, D**).

### 3.2 Weighted gene correlation network analysis and key module selection

**Figure 2:**
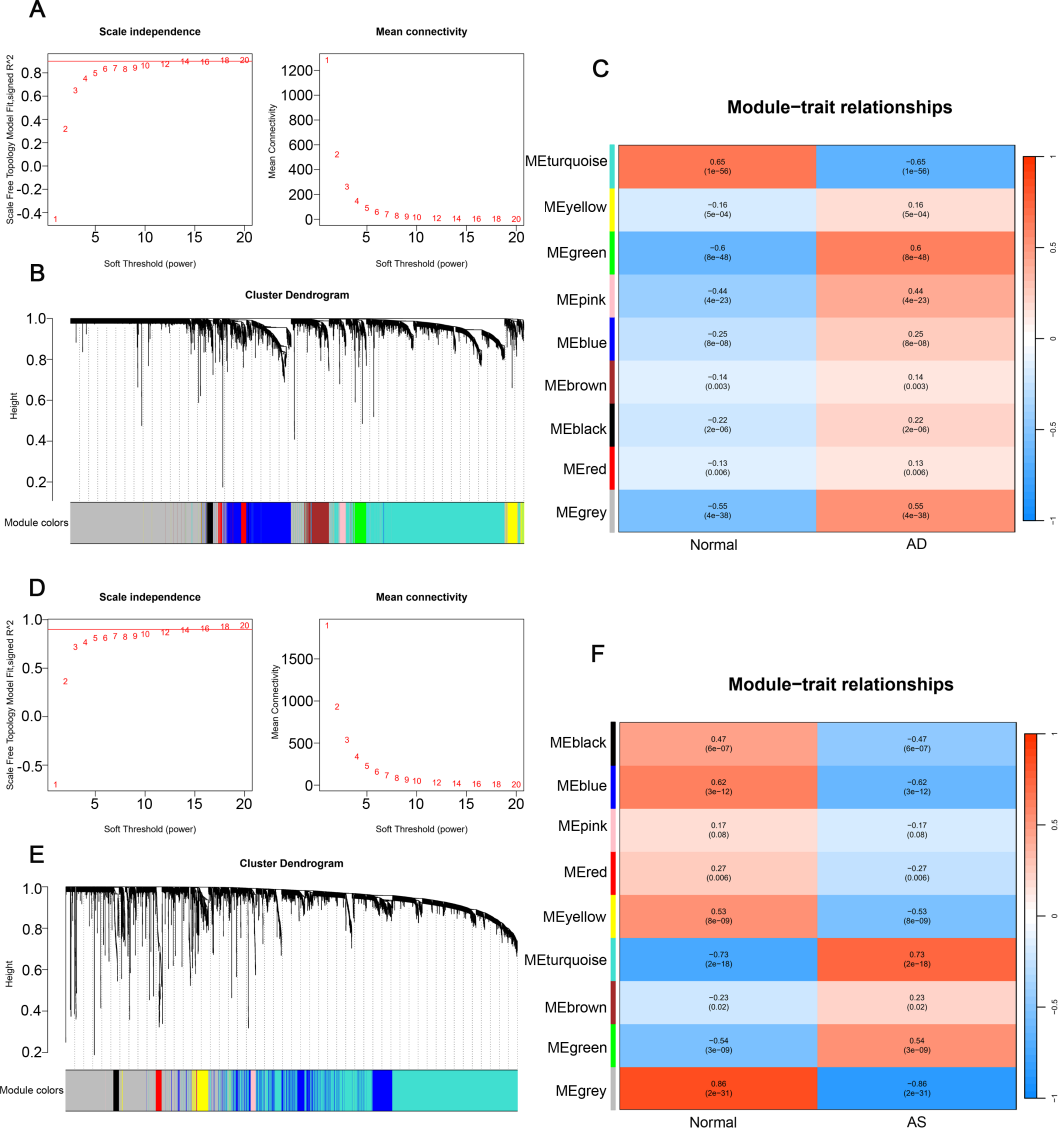
Weighted gene co-expression network analysis. **(A, D)** Determination of soft threshold powers in GSE33000 and GSE100927, R² = 0.90. **(B, E)** Gene cluster trees in GSE33000 and GSE100927. **(C, F)** Relationships between gene modules and traits in disease and normal groups, with numbers in the modules representing correlation coefficients and p-values.

In the AD dataset GSE33000 and AS dataset GSE100927, we employed WGCNA to construct an unsigned co-expression network to identify the gene sets most associated with AD and AS, respectively. For the soft thresholding, we chose a value of 14 for both datasets (**Figures 2A, D**). Under the conditions of a minimum module size of 50 and a merge cut height of 0.25, we generated cluster dendrograms for AD and AS (**Figures 2B, E**). Through clinical correlation analysis, we obtained 9 gene module sets for both diseases (**Figures 2C, F**). Without considering the grey module, we selected the modules MEgreen and Meturquoise (**Supplementary Table 3**), which had the highest positive correlation with AD and AS, respectively, as well as the modules MEturquoise and MEblue (**Supplementary Table 4**), which had the highest negative correlation, for further analysis.

### 3.3 Identification and functional analysis of CGs in AD and AS

**Figure 3:**
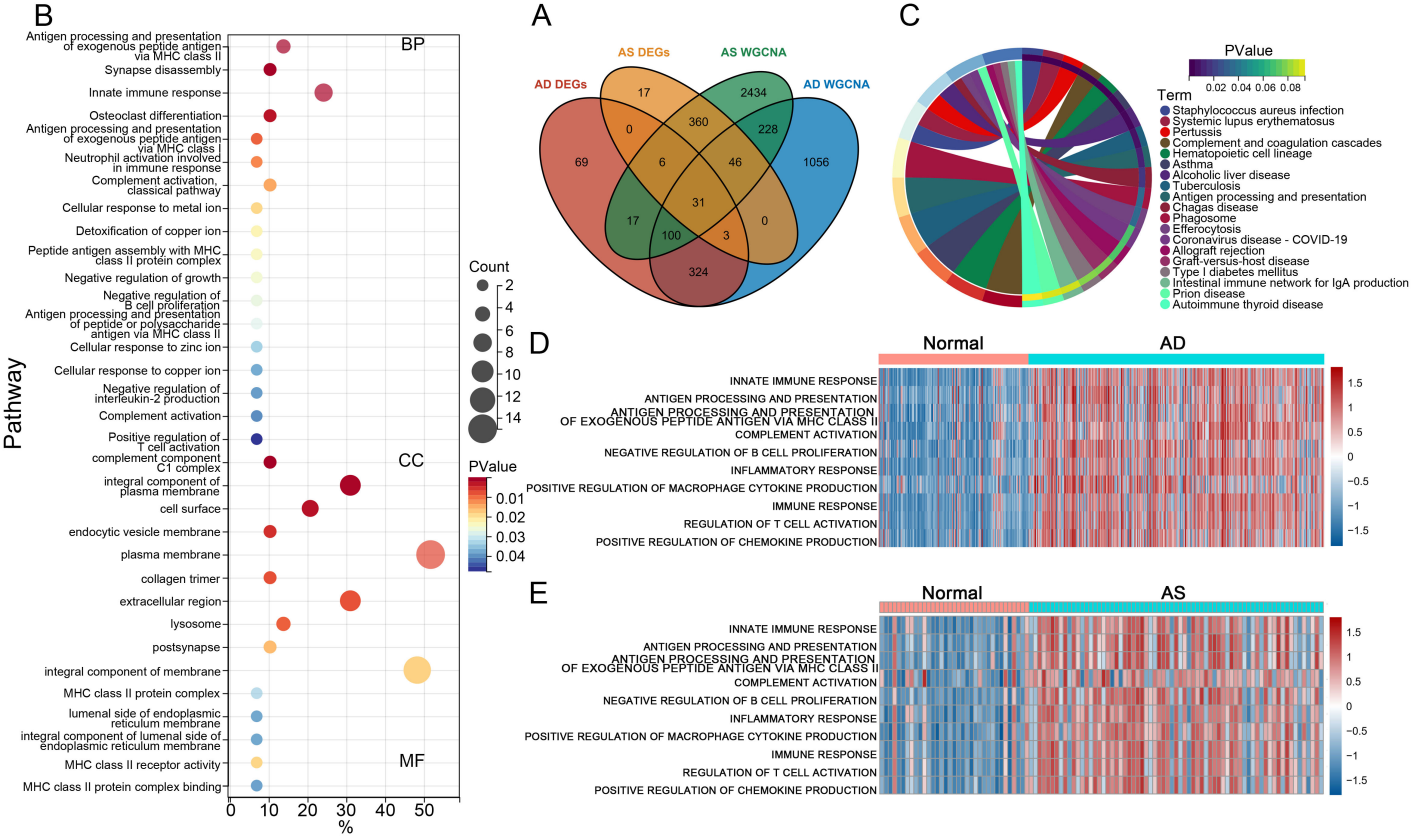
Functional and pathway enrichment analysis. **(A)** Venn diagram illustrates the intersection analysis of DEGs in AD and AS, as well as the gene sets associated with respective traits, determining the crosstalk genes (CGs) for AD and AS. **(B, C)** GO and KEGG enrichment analysis of CGs. **(D, E)** Gene set variation analysis (GSVA) in GSE33000 and GSE100927.

To identify the most closely associated crosstalk gene sets with AD and AS, we performed an intersection analysis between the differentially expressed genes in AD and AS and the relevant gene module sets determined through WGCNA, resulting in 31 CGs for both diseases (**Figure 3A**). Subsequently, we conducted GO functional enrichment analysis for CGs, displaying the results of various aspects sorted by ascending p-values (**Figure 3B**). Biological process analysis demonstrated enriched contents closely related to immune function, such as antigen processing and presentation of exogenous peptide antigen via MHC class II, innate immune response, neutrophil activation involved in immune response, and complement activation classical pathway. In addition, cellular component and molecular function analyses also revealed immune-related contents, including complement components C1 complex and MHC class II receptor activity. Most results from KEGG analysis were also related to the immune system, such as complement and coagulation cascades, antigen processing and presentation, and efferocytosis (**Figure 3C**). Therefore, we inferred a certain correlation between the pathogenesis of AD and AS in the immune system, further validated by GSVA. We selected immune-related biological processes of interest and calculated scores for each patient in the AD dataset GSE33000 and AS dataset GSE100927. The results (**Figures 3D, E**) clearly demonstrated varying degrees of activation of various immune responses in both diseases compared to the normal group.

### 3.4 Identification of the optimal diagnostic genes in CGs through LASSO analysis

**Figure 4:**
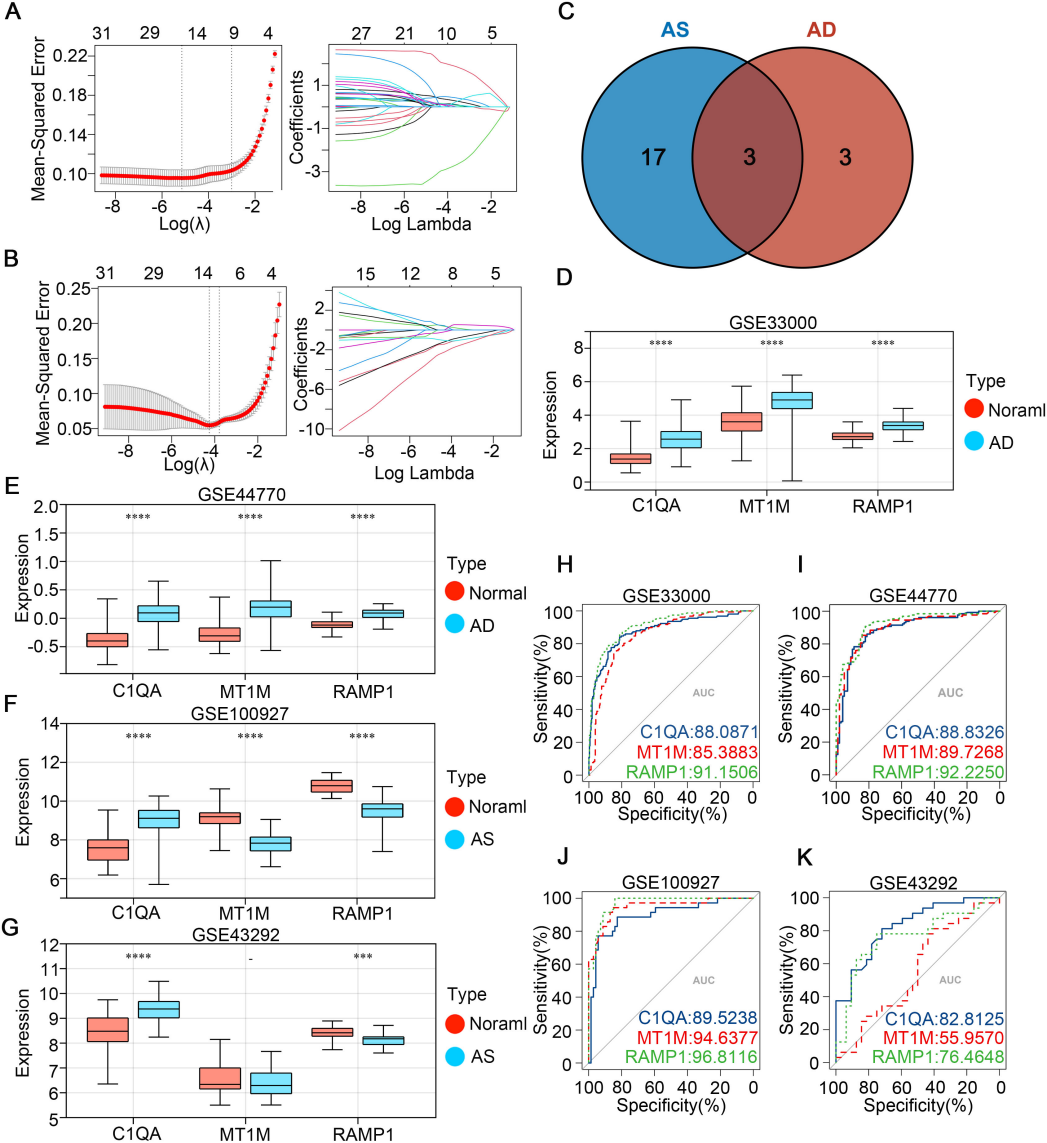
Identifying potential shared disease diagnostic CGs. **(A, B)** LASSO algorithm screening for potential diagnostic CGs in GSE33000 and GSE100927, respectively. **(C)** Venn diagram shows the intersection of potential diagnostic CGs for AD and AS. **(D-G)** Expression patterns of potential shared diagnostic CGs in GSE33000, GSE44770, GSE100927, and GSE43292 for AD and AS. **(H-K)** ROC curve analysis demonstrates the disease prediction ability of shared diagnostic CGs in GSE33000, GSE44770, GSE100927, and GSE43292. ***p < 0.001; ****p < 0.0001.

The CGs identified in the previous step were subjected to LASSO regression analysis using the AD dataset GSE33000 and the AS dataset GSE100927 (**Figures 4A, B**). The λ values were selected as lambda.min for both datasets, followed by intersection analysis. Eventually, we determined three optimal diagnostic genes: C1QA, MT1M, and RAMP1 (**Figure 4C**). To observe the expression patterns of these three genes in the diseases, we analyzed a total of four databases including GSE33000 and GSE100927 as experimental groups for AD and AS, respectively, and GSE44770 and GSE43292 as validation groups for AD and AS, respectively. In the AD dataset, all three genes were found to be highly expressed in the disease (**Figures 4D, F**). In the AS dataset, MT1M showed low expression in the disease, while the remaining genes exhibited high expression (**Figures 4E, G**). Furthermore, to evaluate the predictive accuracy of the three genes for the diseases, we plotted receiver operating characteristic (ROC) curves using the four databases. The area under the curve (AUC) values of the ROC curves were used as indicators of predictive accuracy. The results (**Figures 4H–K**) indicated that in all four databases, the AUC values of the three genes were mostly distributed above 80%, suggesting that the diagnostic genes we identified possess excellent disease prediction capabilities.

### 3.5 Construction and evaluation of AD and AS diagnostic models

**Figure 5:**
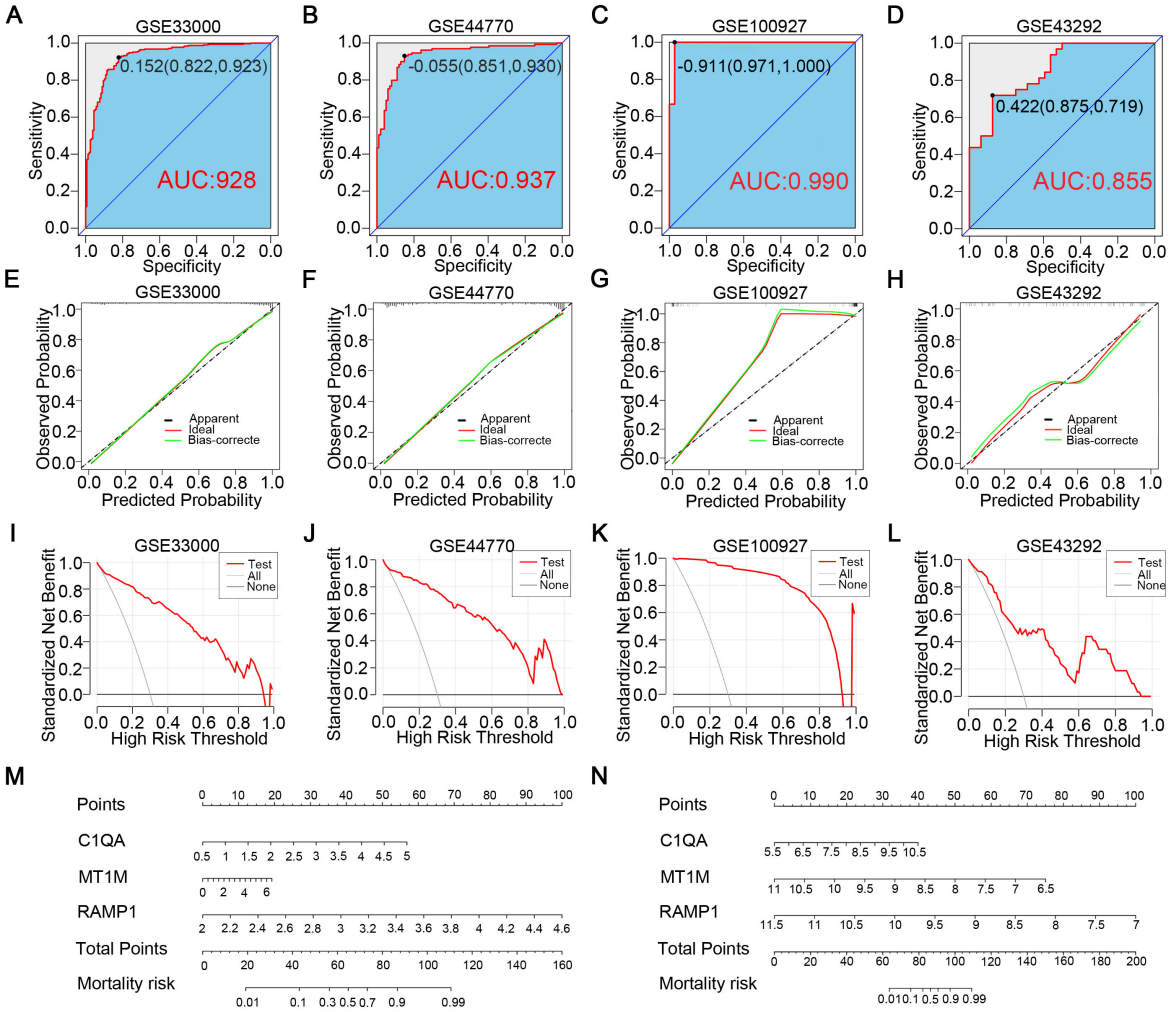
Construction of disease prediction models related to diagnostic CGs. **(A, B)** ROC curves demonstrate the predictive ability of the AD diagnostic model in GSE33000 and GSE44770. **(C, D)** ROC curves demonstrate the predictive ability of the AS diagnostic model in GSE100927 and GSE43292. **(E, F)** Calibration curves of the AD diagnostic model in GSE33000 and GSE44770. **(G, H)** Calibration curves of the AS diagnostic model in GSE100927 and GSE43292. **(I, J)** Clinical decision curves of the AD diagnostic model in GSE33000 and GSE44770. **(K, L)** Clinical decision curves of the AS diagnostic model in GSE100927 and GSE43292. **(M, N)** Disease prediction scoring models established based on diagnostic CGs for AD and AS.

To further ascertain the predictive capabilities of the identified diagnostic genes, we intentionally incorporated the three diagnostic genes into the GSE33000 and GSE100927 databases to respectively construct AD and AS disease prediction models. Utilizing ROC curves, we assessed the disease prediction accuracy of the two models across four databases, and the results (**Figures 5A–D**) indicated that in both the corresponding experimental and validation groups for the two diseases, the AUC values exceeded 0.85. Moreover, in the calibration curves (**Figures 5E–H**), the deviation correction curves for the AD and AS cohorts closely approximated the ideal curve, indicating good model consistency. Additionally, clinical decision curve analysis (DCA) brought deeper clinical significance (**Figures 5I–L**). Across various databases, the net benefits of clinical intervention based on the predicted probabilities from the constructed models were higher within the majority of threshold probability ranges compared to intervening for all or none. Finally, we detailed the characteristics of the AD and AS disease prediction models constructed based on the three diagnostic genes through nomograms (**Figures 5M, N**). In summary, through the aforementioned study, we confirmed the excellent predictive abilities of C1QA, MT1M, and RAMP1 expression as well as the corresponding models in AD and AS.

### 3.6 Selection of hub genes in CGs

**Figure 6:**
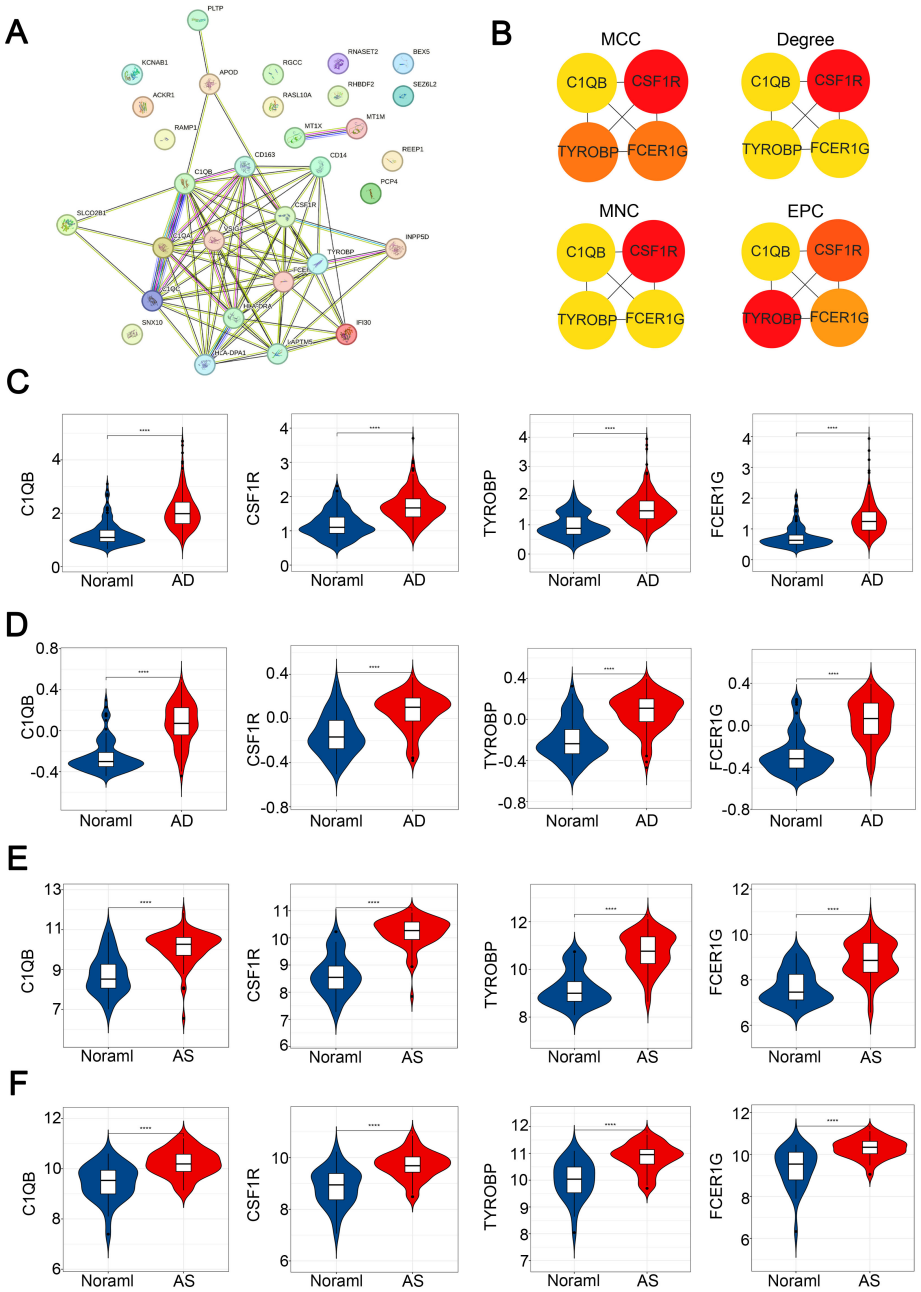
Construction of PPI network and identification of hub CGs. **(A)** PPI network of CGs. **(B)** Hub CGs determined by MCC, Degree, MNC, and EPC algorithms. **(C–F)** Expression patterns of hub CGs in GSE33000, GSE44770, GSE100927, and GSE43292, respectively. ****p < 0.0001.

To identify potential interactions within CGs, we constructed a protein-protein interaction (PPI) network using the STRING database in Cytoscape software, resulting in a network comprising 31 nodes and 79 edges (**Figure 6A**). Simultaneously, we employed four topological analysis methods, including MCC, Degree, MNC, and EPC, to collectively explore hub genes within CGs. According to the results, all four topological analysis methods converged on four common genes: C1QB, CSF1R, TYROBP, and FCER1G (**Figure 6B**). Further analysis of disease expression patterns revealed that these four hub genes exhibited significantly high expression in both the experimental and validation groups for AD and AS (**Figures 6C–F**).

### 3.7 Immune cell infiltration analysis

**Figure 7:**
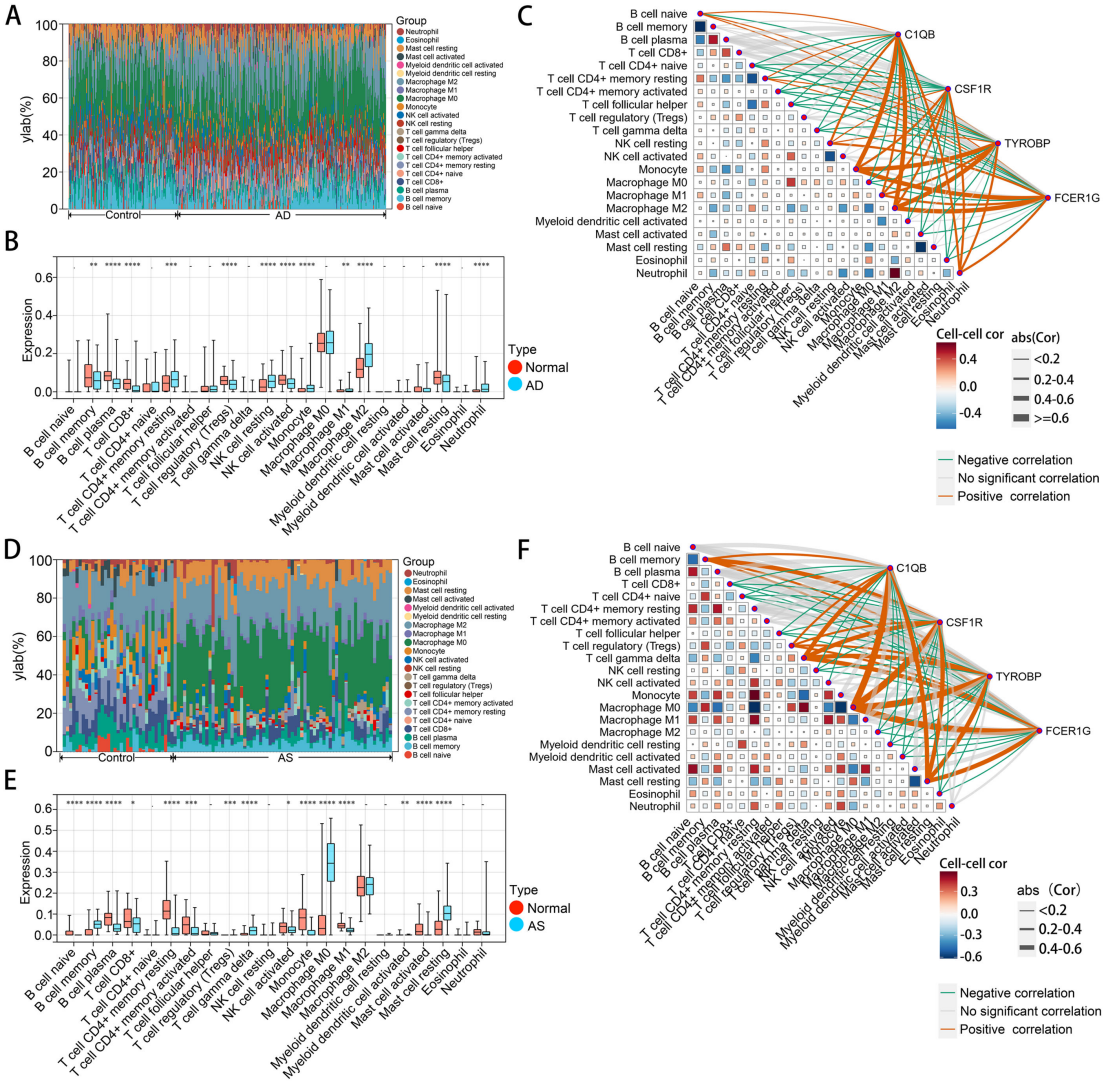
Immune cell infiltration analysis of AD and AS. **(A, D)** Analysis of the proportions of various immune cell infiltrates in GSE33000 and GSE100927 using the CIBERSORT algorithm. **(B, E)** Comparison of levels of various immune cell infiltrates between disease and normal groups in GSE33000 and GSE100927. **(C, F)** Analysis of the correlation between hub CGs and various infiltrating immune cells, as well as among infiltrating immune cells. The upper panel represents AD, and the lower panel represents AS. *p < 0.05; **p < 0.01; ***p < 0.001; ****p < 0.0001.

In order to thoroughly investigate the mechanisms underlying disease pathogenesis, we explored the patterns of immune cell infiltration in AD and AS cohorts. Utilizing the CIBERSORT algorithm, we obtained infiltration scores of various immune cells in the relevant disease tissues. In the GSE33000 dataset, the distribution of immune cells revealed (**Figures 7A, B**) that compared to the normal group, the AD group exhibited a significantly elevated infiltration pattern of M2 macrophages, while B cell memory, B cell plasma, and Mast cell resting showed pronounced decreases in infiltration. In the GSE100927 dataset (**Figures 7D, E**), the AS group displayed an exaggerated increase in infiltration of M0 macrophages compared to the normal group, while B cell plasma, T cell CD4+ memory resting, and Monocyte showed noticeable decreases in infiltration. Correlation analysis (**Figures 7C, F**) demonstrated a high consistency between the identified hub genes in CGs and the relationship with immune cells observed in both diseases’ immune infiltration characteristics. This confirms the inseparable relationship between the expression of the hub genes identified earlier and the development of both diseases.

### 3.8 Construction of hub gene interaction networks

**Figure 8:**
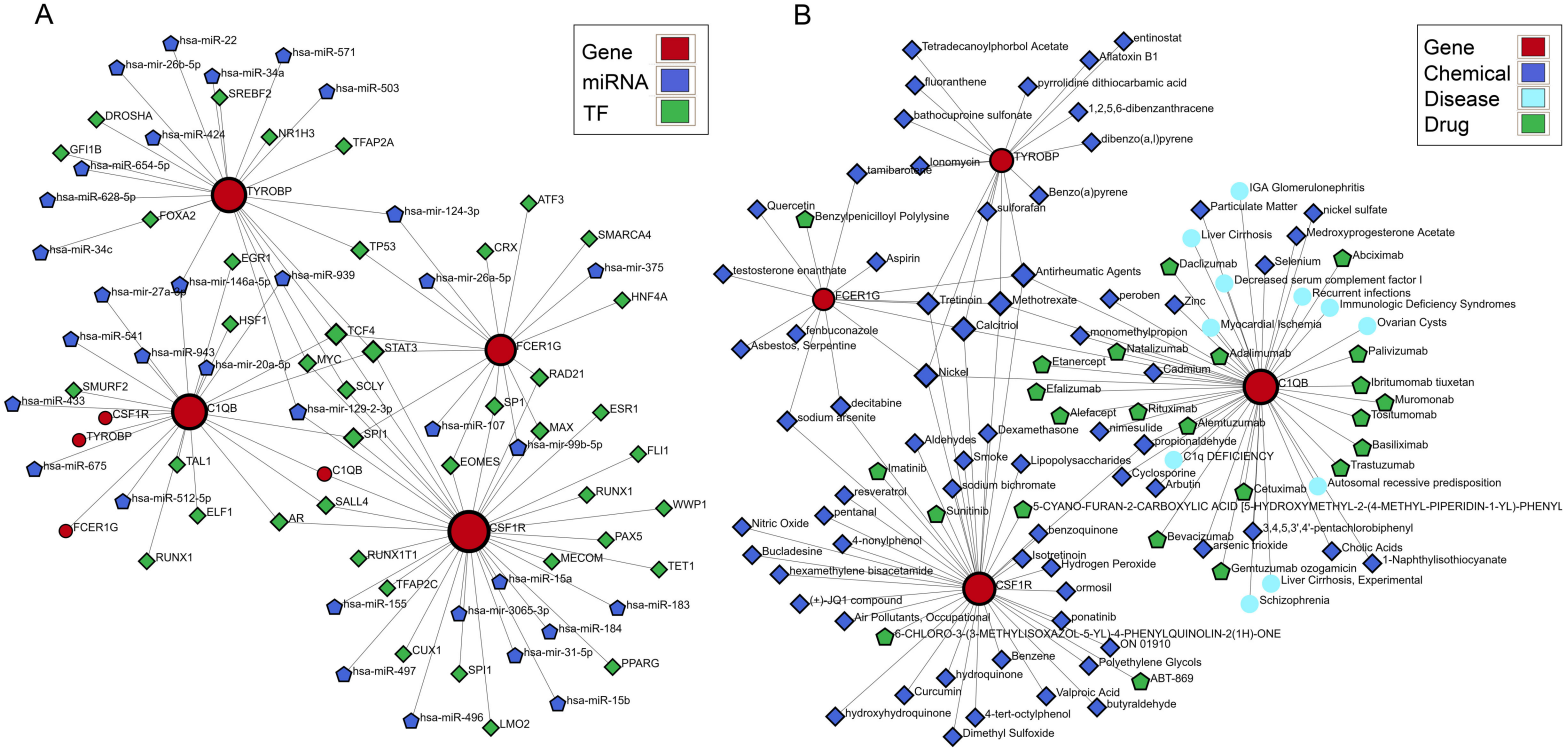
Building the interacting network of hub CGs. **(A)** Gene-miRNA interaction network, TF-gene interaction network, and TF-miRNA co-regulatory network. **(B)** Protein-chemical association, protein-drug association, and gene-disease association networks.

To confirm the upstream and downstream interactions of hub genes and their associated content, we separately constructed regulatory networks of hub genes and associated networks of diseases, drugs, and chemicals. The gene regulatory network includes gene-miRNA interaction network, TF-gene interaction network, and TF-miRNA co-regulation network (**Figure 8A**). It can be observed that C1QB occupies a central position in hub gene interactions, with regulatory factors TCF4, MYC, STAT3, and SCLY playing a co-regulatory role in hub genes. has-mir-146a-5p, has-mir-124-3p, has-mir-129-2-3p, and has-mir-99b-5p are important miRNAs in the hub gene network. In the protein-chemical, protein-drug, and gene-disease associated networks (**Figure 8B**), associated diseases and drugs mainly focus on the action of C1QB, while chemicals such as Nickel, Tretinoin, Calcitriol, Methotrexate, and Antirheumatic Agents are significant relevant substances. These findings demonstrate closely associated networks of actions with hub genes in both diseases.

### 3.9 Identification and characteristic analysis of subtypes in two disease types based on CGs

**Figure 9:**
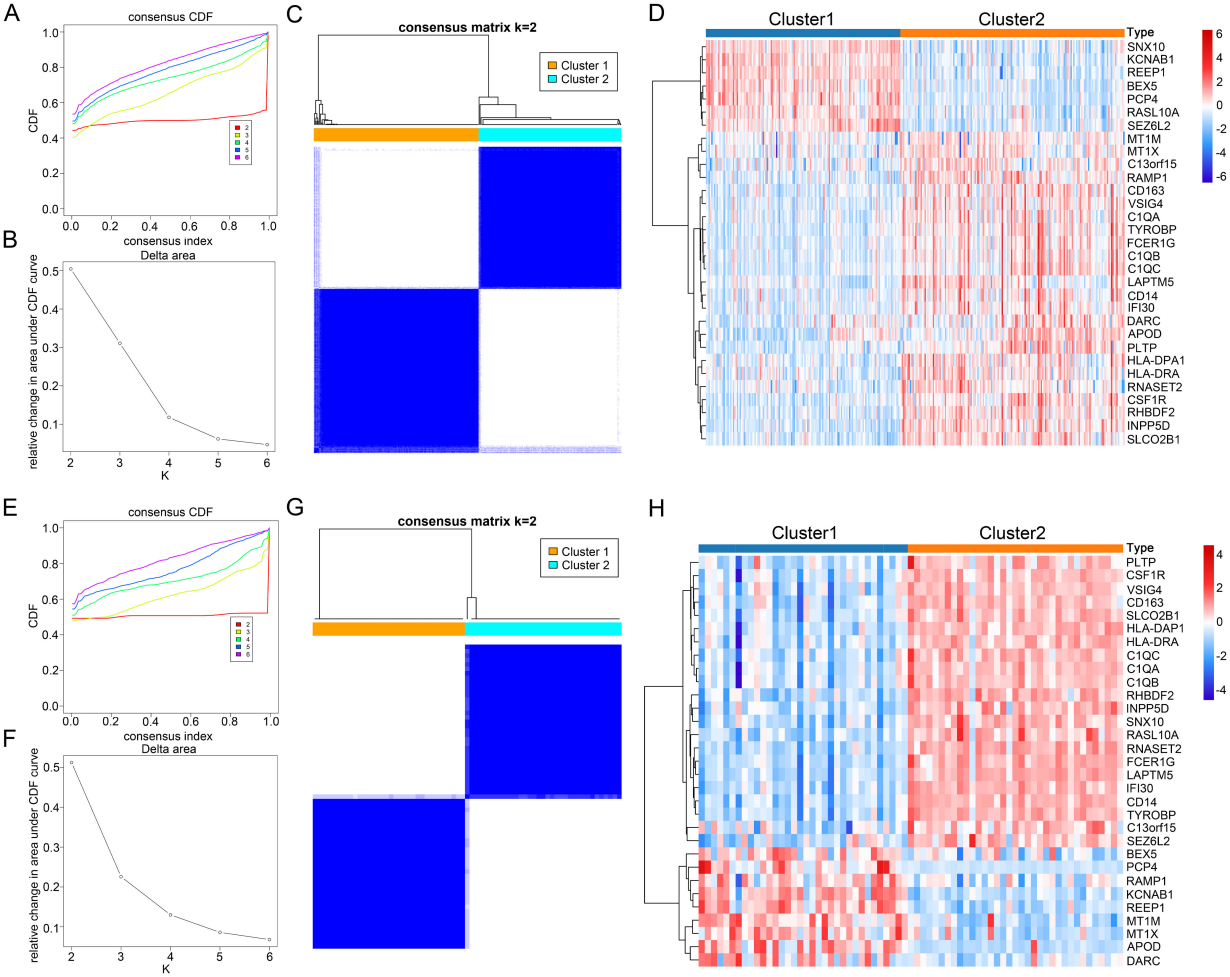
Identification of AD and AS disease subtypes related to consensus clustering of CGs. **(A, B)** Changes in the values of CDF and the corresponding area under the CDF curve in GSE33000 for k = 2-6. **(E, F)** Changes in the values of CDF and the corresponding area under the CDF curve in GSE100927 for k = 2-6. **(C)** Consensus matrix heatmap of AD subtype at k = 2. **(G)** Consensus matrix heatmap of AS subtype at k = 2. **(D)** Expression heatmap of different subtype CGs in AD cohort. **(H)** Expression heatmap of different subtype CGs in AS cohort.

**Figure 10:**
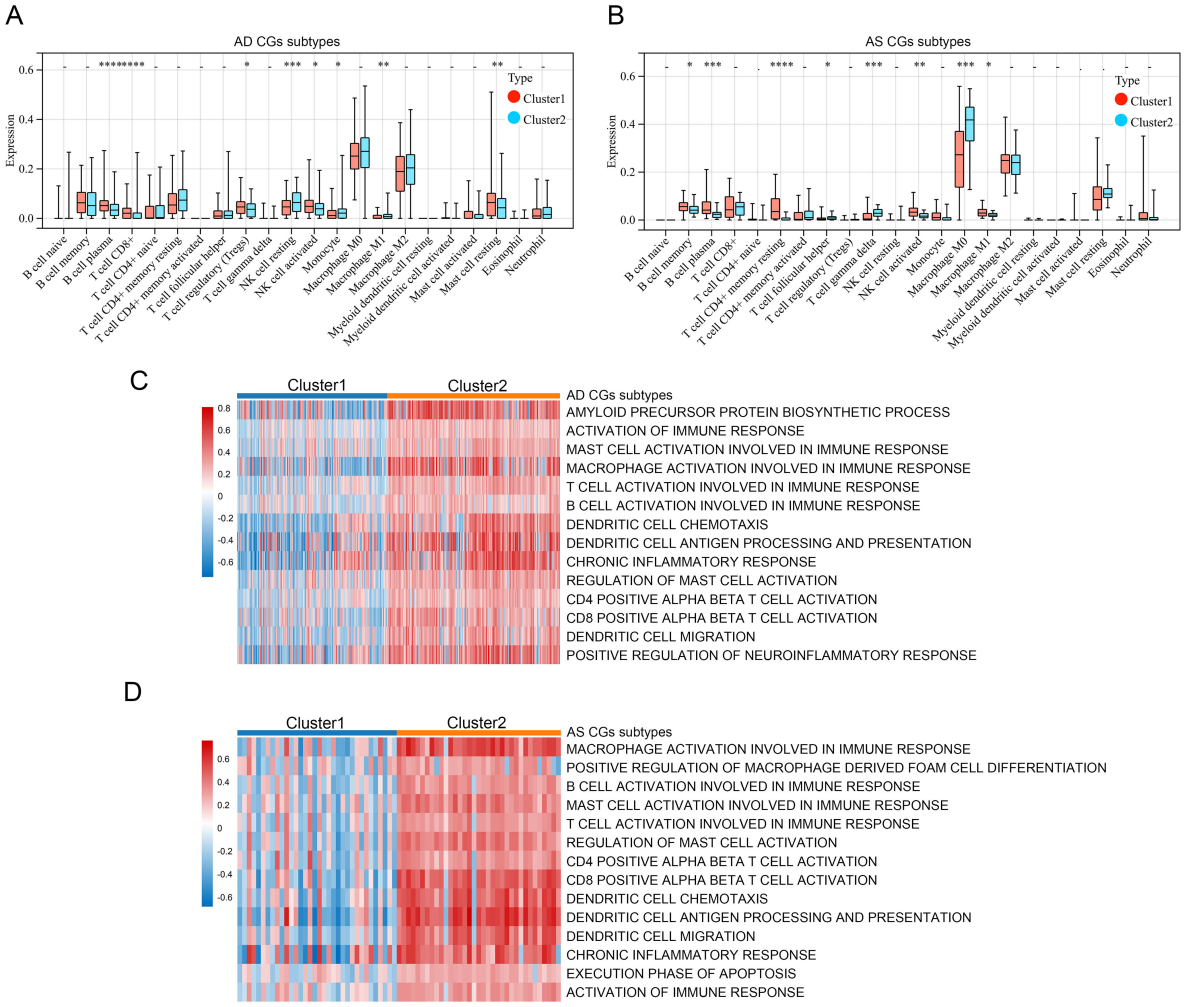
Relevant features of AD and AS disease subtypes. **(A, B)** Analysis of immune cell infiltration levels of C1 and C2 subtypes in AD and AS using the CIBERSORT algorithm. **(C, D)** GSVA demonstrates the enrichment status of different functional pathways in C1 and C2 subtypes. *p < 0.05; **p < 0.01; ***p < 0.001; ****p < 0.0001.

Finally, to comprehensively understand the impact of CGs expression on AD and AS, we identified subtypes of CGs through consensus clustering analysis for each disease. Consensus clustering analysis of CGs expression profiles in AD identified two subtypes, C1 and C2, among AD patients in GSE33000 (**Figures 9A–C**). Similarly, consensus clustering analysis of CGs expression profiles in AS also identified two subtypes, C1 and C2, among AS patients in GSE100927 (**Figures 9E–G**). Heatmaps were generated to illustrate the expression patterns of CGs in the two subtypes of AD and AS (**Figures 9D, H**). Subsequently, we performed immune infiltration analysis and enrichment score calculation of disease-related pathways using the CIBERSORT algorithm and GSVA algorithm for the subtypes of both diseases. Results indicated that, compared to the C1 subtype, the C2 subtype in both AD and AS largely exhibited expression patterns of immune cell infiltration consistent with the inherent immune infiltration characteristics of the diseases, particularly in macrophage infiltration features (**Figures 10A, B**). Calculation of enrichment scores (**Figures 10C, D**) revealed a significant immune activation state in the C2 subgroups of both diseases, including activation of various immune cells and regulation of inflammatory cells. Furthermore, in their respective disease mechanisms, pathways such as amyloid precursor protein biosynthesis and positive regulation of neuroinflammatory responses in AD, as well as positive regulation of macrophage-derived foam cell differentiation in AS, showed significant activation in the C2 compared to the C1 subtype. Overall, these consistent pieces of evidence suggest a key role of CGs in the pathogenesis of AD and AS, indicating important connections between AD and AS at their core disease level.

## 4 Discussion

Alzheimer’s disease (AD) and atherosclerosis (AS), as two major diseases in the world today, pose significant challenges to human society due to their progressive courses and increasing prevalence (43, 44). Although they are different diseases, there are important associations and interactions between them. AD patients often have a higher risk of cardiovascular diseases, such as hypertension, high cholesterol, and diabetes, which may be related to the development of AS (45). AS may accelerate the progression of AD by damaging the vascular endothelium, allowing harmful substances to enter the brain (46). Additionally, some pathophysiological changes in AD, such as amyloid plaques and abnormal tau protein deposition within neurons, may be associated with blood supply insufficiency and disrupted neuronal energy metabolism caused by AS (47). It is important to note that both diseases are related to chronic low-grade inflammation caused by aging, and shared inflammatory responses and immune dysregulation mechanisms may be key links between them (34). Given the intricate interaction mechanisms between them, a thorough understanding of their potential comorbid mechanisms is crucial for the prevention of both diseases.

This study identified 31 CGs between AD and AS, and functional analysis results showed significantly enhanced immune and inflammatory responses in both diseases compared to healthy patients. Previous research on neurodegenerative diseases has shown that inflammation is not only a result of these diseases but also a key participant in the process (48). In the case of AS, chronic inflammation of the arterial wall has long been considered a key cause of its pathogenesis (33). Nowadays, there has been significant progress in understanding the inflammatory and immune responses in AD and AS, and targeted treatments for long-term immune and inflammatory responses have gained increasing consensus (49, 50). In our subsequent research, three biomarkers (C1QA, MT1M, and RAMP1) were finally identified and demonstrated good diagnostic capabilities for both diseases. C1q is an important component of the complement system, playing a crucial role in maintaining immune homeostasis (51). The A-chain peptide of serum subcomponent C1q is encoded by the C1QA gene, and research has shown that C1QA may promote synaptic loss and be associated with progressive neurodegeneration (52, 53). Furthermore, clinical research results confirm the involvement of C1q in the development of atherosclerosis, plaque instability, and obstructive coronary artery disease (54, 55). Metallothionein 1M (MT1M) is a zinc-binding protein belonging to the metallothionein family, rich in cysteine, and plays an important role in regulating oxidative stress (56, 57). It is widely expressed in various tissues and protects cells from oxidative stress damage by scavenging free radicals and releasing zinc into the cytoplasm (58, 59). Interestingly, previous studies have also shown the involvement of MT1M in the inflammatory process, as pro-inflammatory factors can increase MT1M expression (60). RAMP1 belongs to the receptor activity-modifying protein (RAMP) family, best known for its role in modulating the activity of the calcitonin receptor (CLR), which has significant implications in the treatment of migraines (61, 62). Recently, there have also been reports of a close association between RAMP1 and tumors (63). Although research on RAMP1 is limited, increasing evidence suggests that RAMP1 plays important roles in the nervous, immune, endocrine, and circulatory systems, making it a potential new hotspot for disease development (64–67). Subsequently, the disease risk prediction model based on C1QA, MT1M, and RAMP1 was thoroughly validated using a verification database, suggesting that these three biomarkers are worthy of further in-depth study.

In studying the CGs that play a central role, we incorporated four topological analysis methods to jointly identify four hub CGs (C1QB, CSF1R, TYROBP, and FCER1G). C1QB is a polypeptide chain of the serum complement subcomponent C1q, and the effects of C1q on the nervous system and atherosclerosis have already been described earlier. CSF1R is a transmembrane receptor that initiates signal transduction pathways within cells by binding with ligands CSF-1 and IL-34 (68–70). Studies have indicated that CSF1R signaling is involved in regulating the activity of immune cells, promoting cell survival, proliferation, and differentiation, especially in macrophages, microglia, osteoclasts, and bone marrow dendritic cells (71). Moreover, excessive CSF1R signaling can sustain microglial activation, leading to the occurrence of chronic neuroinflammation and subsequent neurodegenerative changes (72, 73). TYROBP, also known as DAP12, is a transmembrane adaptor protein widely expressed on immune cells, serving as a downstream adapter and presumed signaling partner for various receptors associated with AD, notorious for its role (74). Additionally, research suggests that TYROBP can promote lipid deposition and plaque inflammation during the AS process (75). FCER1G has been identified as a marker for human aging and neurodegenerative diseases in microglial cells (76). Recent studies have also confirmed the significant role of FCER1G in promoting immune cell infiltration into atherosclerotic plaques and intraplaque hemorrhage (77). Overall, these four hub CGs are intricately linked to both AD and AS.

The results of immune cell infiltration analysis show a significant enrichment of M2 macrophages in the AD environment. It is believed that the recruitment of peripheral macrophages to the central nervous system is likely a potential therapeutic target for AD (78). Currently, activated macrophages are mainly divided into two subtypes: M1 and M2. M1 macrophages primarily promote inflammatory responses, while M2 macrophages mainly inhibit inflammatory responses (79). An interesting study found that in AD model rats, transplantation of M2 macrophages could reduce intracranial inflammatory responses, decrease neuronal loss, and improve cognitive dysfunction, suggesting that M2 macrophages have a protective role in AD (80). The enrichment of M2 macrophages in the brains of AD patients is hypothesized to be a form of self-protection by the body. How to utilize this phenomenon may be worth further in-depth research by future scholars. In the AS environment, M0 macrophages exhibit more significant infiltration. This aligns with the pathogenesis of AS, where macrophages engulf modified low-density lipoprotein particles and form foam cells (a hallmark of atherosclerosis), leading to the formation of early atherosclerotic lesions (81, 82). Nowadays, since lipid-lowering therapy cannot completely halt the progression of AS and macrophage polarization is involved in various stages of atherosclerosis, an increasing number of AS treatment strategies are focusing on targeting macrophages (83–85).

The construction of gene interaction networks provides a more detailed illustration of the regulatory mechanisms of hub CGs and their associated diseases, drugs, and chemicals, enhancing our understanding of disease onset and aiding in the development of treatment strategies. The regulatory factors TCF4, MYC, STAT3, and SCLY play the most extensive co-regulatory roles in CGs. TCF4, a member of the helix-loop-helix (HLH) protein family, is expressed in various cell types and tissues throughout the body (86). Research has shown that TCF4 is a key regulator of neural function and is closely associated with neurodevelopmental disorders such as intellectual disability and schizophrenia (87, 88). Recent studies have also indicated that TCF4 influences IL-17RA/IL-17RE signaling, which is involved in inflammatory feedback loops (89). Current research on MYC primarily focuses on its role in cancer (90). MYC is a super-transcription factor encoded by the MYC gene located on chromosome 8q24.21, playing a crucial role in cell growth, proliferation, and apoptosis (91, 92). STAT3’s mechanisms have been confirmed in Alzheimer’s disease (AD) model mice, where inhibiting STAT3 expression improves pathological and behavioral abnormalities (93). Additionally, STAT3 has been shown to promote the progression of ankylosing spondylitis (AS) through mechanisms such as interference with the Akt/mTOR signaling cascade and pyroptosis (94–96). Studies on SCLY indicate its involvement in selenium methionine metabolism and its potential role in oxidative stress and cellular protection (97). Chemical compounds such as Nickel, Tretinoin, Calcitriol, Methotrexate, and Antirheumatic Agents have been closely linked to pivotal CGs in analyses. These compounds may play significant roles in future research.

Consensus clustering analysis based on CGs revealed two distinct subtypes of immune and inflammatory activation intensities within AD and AS. This finding highlights the crucial role of CGs in the pathogenesis of AD and AS and underscores the importance of immune and inflammatory dysregulation in the occurrence and progression of these diseases.

Despite the aforementioned analyses, this study still has certain limitations. Utilizing online databases, we analyzed and identified CGs for both AD and AS; however, the database itself may not be comprehensive, and we lack experimental validation in aspects such as gene function and immune infiltration. Additionally, disease prediction models and disease subtypes built based on CGs may require further clinical validation before entering formal applications. Therefore, in the future, we hope to see more researchers joining this study.

## 5 Conclusion

In recent years, age-related diseases have garnered increasing attention. We are dedicated to filling the gap in understanding the interaction mechanisms between Alzheimer’s disease (AD) and atherosclerosis (AS) and have identified crosstalk genes between AD and AS. C1QA, MT1M, and RAMP1 have been identified as potential diagnostic biomarkers, and predictive models for both diseases have been constructed based on these genes. Additionally, C1QB, CSF1R, TYROBP, and FCER1G have been recognized as key genes in the crosstalk between AD and AS, showing close associations with immune cells in immune infiltration analysis. By establishing a gene interaction network, we have more clearly demonstrated the regulatory mechanisms and related functions of these key genes. Overall, we have identified seven important crosstalk genes, which have been confirmed in extensive studies to play significant roles in the immune process. However, the specific roles of these genes in AD and AS still require further research. In the future, these findings are expected to provide new clues for exploring targeted therapeutic approaches for both diseases.

## Data Availability

The datasets analyzed for this study can be found in the GEO database; GSE33000, GSE100927, GSE44770, and GSE43292.

## Supplementary material

Supplementary Table 1 DEGs in the expression matrix of GSE33000.

Supplementary Table 2 DEGs in the expression matrix of GSE100927.

Supplementary Table 3 Identification of the MEgreen module most positively correlated with AD and the MEturquoise module most positively correlated with AS by WGCNA.

Supplementary Table 4 Identification of the MEturquoise module most negatively correlated with AD and the MEblue module most negatively correlated with AS by WGCNA
